# Dendritic cells derived exosomes migration to spleen and induction of inflammation are regulated by CCR7

**DOI:** 10.1038/srep42996

**Published:** 2017-02-22

**Authors:** Gao Wei, Yuan Jie, Liu Haibo, Wu Chaoneng, Huang Dong, Zhu Jianbing, Guo Junjie, Ma Leilei, Shi Hongtao, Zou Yunzeng, Ge Junbo

**Affiliations:** 1 Department of Cardiology, Shanghai Institute of Cardiovascular Diseases, Zhongshan Hospital, Fudan University, Shanghai, China; 2 Department of Cardiology, East Hospital, Tongji University School of Medicine, Shanghai, China

## Abstract

Mature dendritic cells (DCs) home to secondary lymphoid organs through CC chemokine receptor 7 (CCR7). Exosomes derived from DCs (DC-exos) are reported to migrate to spleen and induce inflammation *in vivo*. In this study, we demonstrated that mature bone marrow DC-exos can activate immature DC and T cells *in vitro*. Then we intravenously injected DC-exos into C57BL/6 mice, observing that mature DC-exos accumulated more in spleen than immature DC-exos. These DC-exos in spleen could be uptaken by splenetic DCs and T cells and induce an inflammatory response. We further showed that the increased accumulation of mature DC-exos in spleen was regulated by CCR7, whose reduction led to a decrease of accumulation in spleen and attenuated inflammatory response in serum. These data provide us a new perspective to comprehensively understand exosomes, which might inherit some special functions from their parent cells and exert these functions *in vivo*.

Dendritic cells (DCs) are innate immune cells that play a unique role as central orchestrators of the immune response. They express pattern recognition receptors, such as toll-like receptors (TLRs), to sense pathogens, lipids, and other biomolecules. Along with macrophages, DCs also are a class of professional antigen-presenting cells (APCs), which express high levels of the major histocompatibility complex class II (MHC-II) molecule and link innate and adaptive immune responses by presenting antigens to T-cells. Under normal condition, DCs reside in relatively low numbers in the peripheral tissues and in greater numbers within secondary lymphoid tissues, including the lymph nodes and spleen. When activated by infectious or inflammatory stimulus, DCs undergo maturation characterized by the upregulated expression of MHC-II molecules and co-stimulatory molecules such as CD80, CD83 and CD86, as well as CC-chemokine receptor 7 (CCR7)^1^. An important *in vivo* feature of DCs is homing and migration. They migrate from the blood to the tissues and then capture antigens. Then, following inflammatory stimuli, they undergo mature and leave the tissues and move to the draining lymphoid organs where they prime naive T cells. CCR7 and its ligands are essentially involved in homing of DCs to the lymph nodes and spleen^2^,^3^,^4^.

Exosomes are a subset of extracellular vesicles released by almost all kinds of cell types. It has been reported that exosomes contain a variety of biological components, including membrane proteins, lipids, RNA and even DNA^5^. Upon being released from cells, exosomes can distribute in biological fluids and be up-taken by the same or a different type of cells, which then interact with exosomes and present biological functions^5^. Previous studies demonstrated that DCs-derived exosomes (DC-exos) can exert biological effects via membrane proteins or inner contained miRNAs^6^,^7^,^8^. Specifically, DC-exos can migrate to spleen, deliver miRNAs to immune cells in spleen and modulate inflammatory response to endotoxin^8^. Previous research, based on spectrometry study, showed that mature DC-exos harbored a large population of proteins, including MHC-II and co-stimulatory molecules, with which mature DC-exos induce immune responses^9^,^10^,^11^. A recent study found that after intravenous injection into mice, DC-exos showed increased accumulation in spleen compared with exosomes derived from other sources^12^.

Thus we postulated that CCR7 might be on the DC-exos membrane and contribute to DC-exos migration to spleen and induction of inflammation. Here in the present study, we firstly demonstrated that mature DC-exos significantly induced immature DCs and T cells to secrete elevated cytokines. Then we showed that, after intravenous injection, mature DC-exos accumulated more in spleen compared with immature DC-exos and induced a significant inflammatory response in mice. The expression of CCR7 in both immature DC-exos and mature DC-exos were detected and we found that mature DC-exos contain more CCR7. When we down-regulated CCR7 in mature DC-exos, we observed a decrease of exosomes accumulation in spleen and attenuated inflammatory response in serum. Our results, along with previous studies, provide a comprehensive understanding of mature DCs derived exosomes, which can migrate to lymphoid organs and induce immune responses just like their parent DCs.

## Materials and Methods

### Ethics

All of the animal studies were performed in accordance with the Council of Europe Convention for the protection of vertebrate animals used for experimental and other scientific purposes with the approval of the National and Local Animal Care Committee. Animal care and treatment complied with the standards approved by the Institutional Review Board of Zhongshan Hospital at Fudan University and the Shanghai Institutes for Biological Sciences-CAS (A5894-01).

### DCs culture

As described previously, bone marrow DCs were from C57BL/6 mice^13^. To eliminate the interference of exosomes from fetal bovine serum, we used a non-serum medium, X-VIVO 15 (LONZA), to culture DCs^14^. Briefly, bone marrow progenitors were washed out and cultured in medium X-VIVO containing 10 ng/ml granulocyte-macrophage colony-stimulating factor and 1 ng/ml IL-4 (PeproTech). Non-adherent cells were gently washed out at 48 h. The remaining clusters were cultured and the medium was changed every other day. On day 7, the cells were mostly immature DCs and they can be used for study. To induce these DCs mature, we treated them with LPS (5ug/ml) for 24 hours.

For the purpose of exosomes isolation from culture medium, immature or mature DCs were washed twice with PBS and replaced with fresh medium. After another 36–48 hours of continuous culturing, the culture medium was collected for exosomes isolation according to manufacturer’s protocol with some modifications.

### DCs transfection

To transfect siRNA CCR7 into DCs, we used transfection reagents (riboFECT™ CP, Ribobio, China) according to manufacturer’s protocol. Briefly, DCs were transfected with siRNA CCR7 at a concentration of 50 nM for 24 hours and treated with LPS or PBS for 12 hours. Then the DCs were washed twice with PBS and replaced with fresh medium. After another 36 hours of continuous culturing, the culture medium was collected for exosomes isolation.

### Exosomes isolation, analysis, uptake and immunofluorescence

Exosomes were precipitated by using exosome precipitation solution (Exo-Quick; System Bioscience) following the manufacturer’s instructions with some modifications^15^. Briefly, the culture medium was harvested at 3000 × g for 15 min and then underwent a centrifugation at 10000 g for 30 minutes to eliminate cell debris. The obtained supernatant was then filtrated with 0.22 um filter in order to further eliminate cell debris and large particles. ExoQuick was added to the medium at a ratio of 1:5 and the mixed solution was placed at 4 degree centigrade over night. Then we centrifugated the solution at 1500 g for 30 minutes, resuspended the exosomes pallet with PBS and stored them at −80 °C for subsequent studies.

The ultrastructure and size distribution were analysed by transmission electron microscopy and Nanosight (Malvern), respectively. Protein markers, CD63, Alix, TSG101, Calnexin and CCR-7 were determined by immunoblotting.

To determine whether DCs can uptake DC-exos, we stained exosomes with PKH67 (Sigma) according to protocols previously reported^16^. Exosomes diluted in PBS were added to 0.5 ml Diluent C. In parallel, 4 μl PKH67 dye was added to 0.5 ml Diluent C and incubated with the exosome solution for 4 minutes at room temperature. In order to bind excess dye, 2 ml 0.5% BSA/PBS was added. The labeled exosomes were washed at 100,000 g for 1 hour, and the exosome pellet was suspended with PBS and used for uptake experiments. We then co-culture these labeled exosomes with fresh immature DCs or inject them into mice. A total of 100 μL (20 ug) DC-exos were co-cultured with 10^5^ DCs in a final volume of 250 mL or injected to every single mouse. After indicated time of co-culture, we stained DCs with DAPI (Sigma) and observed them with confocal microscopy. After 24 hours of injection, we excised the spleen from mice and serial cryostat sections (6 μm) were prepared using a Lab-Tek tissue processor (Leica). The samples were stained with CD11c/CD4 (Abcam) and DAPI and observed with confocal microscopy.

CD4+ T cells were purchased from Allcells (Shanghai, China). To determine whether T cells can uptake DC-exos *in vitro*, we stained mouse spleen CD4+ T cells with PKH67, and the DC-exos with PKH26 (Sigma-Aldrich). A total of 10^5^ CD4 + T cells were cultured with 100 μL (20 ug) DC-exos in a final volume of 250 mL for 24 h. The cells were fixed with 2% paraformaldehyde and were analyzed by flowcytometry for quantification.

### Western blotting of exosomal protein

The exosome pallet was lysed in RIPA buffer supplemented with complete protease inhibitor cocktail tablets (Roche). Lysates were separated by SDS-PAGE gels, transferred to PVDF membranes (Bio-Rad), and incubated with the relevant antibodies as indicated. Antibody against CD63 was purchased from Santa Cruz Biotechnology, Alix was purchased from Cell Signaling Technology and TSG101, Calnexin and CCR7 were purchased from Abcam.

### Real-Time PCR

Total RNA was extracted using TRIzol reagent (Sangon) from spleen tissues or DCs. ReverTra Ace qPCR RT Kit (TOYOBO) was used to generate cDNA from mRNA and SYBR Premix Ex Taq (Takara) was used for real-time qPCR with the ABI 7500 Real-time PCR system following the manufacturer’s instructions. The relative expression levels of the genes were normalized to that of GAPDH by using the 2^−ΔΔCt^ cycle threshold method.

### Elisa

The serum of mice was analyzed for IL-6 and TNF-α with ELISA kits (Anogen, Ontario, Canada) according to manufacturer’s instruction. The supernatants of CD4+ T cell were analyzed using a mouse CBA inflammation kit (BD, USA) according to manufacturer’s instruction.

### Animal experiments

C57BL/6 J mice were used for animal studies. To investigate the distribution of DC-exos in mice, we stained exosomes (20 ug) with DiR (KeyGEN, Nanjing, China) and injected them into mice through tail vein. For imaging, IVIS Spectrum (Perkin Elmer) was used. Here, both live (isoflurane sedated) mice and organs were imaged. The live mice were consecutively imaged at indicated time. The organs were harvested and imaged 4 hours after injection. To determine the effect of mature DC-exos on mice, we took the mice blood by removing eyeball 24 hours after injection. After 30 minutes’ standing at room temperature, the blood samples were centrifuged at 5000 rpm for 30 minutes. Then the upper serum was collected for Elisa detection.

### Statistical analysis

Data are expressed as the means ± S.D. Student t test was used to determine statistical significance between the groups. A P < 0.05 was considered significant.

## Results

### Successful isolation of exosomes from DCs culture medium

**Figure 1:**
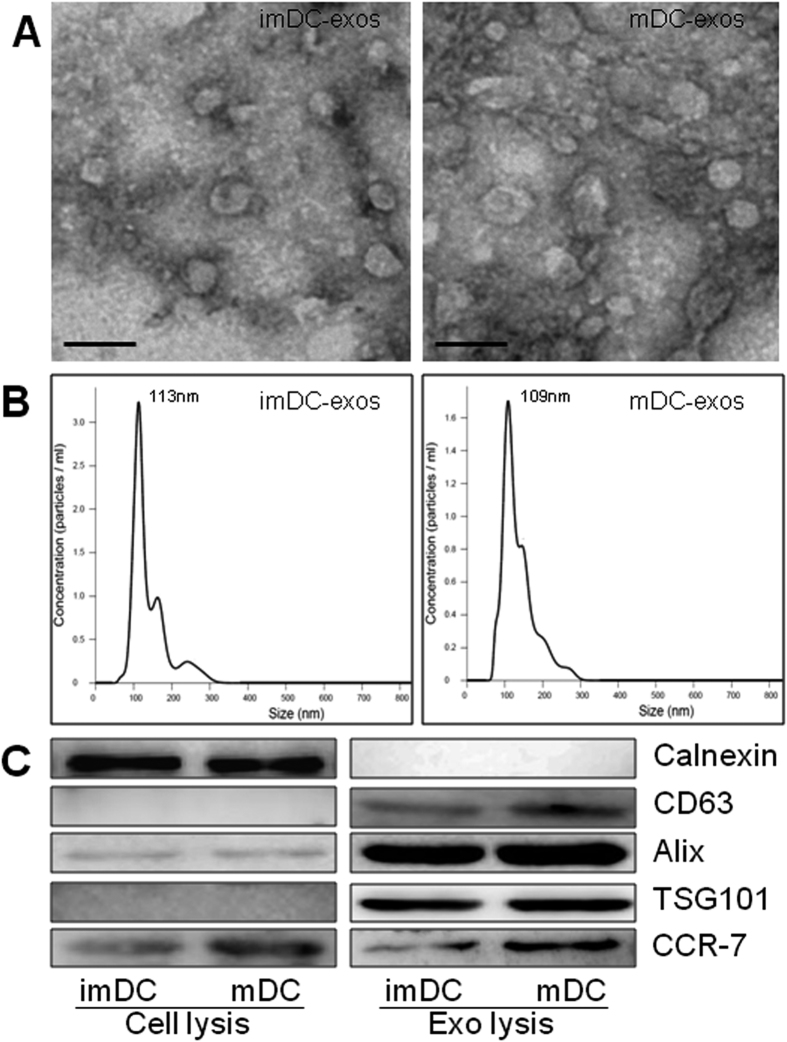
Successful isolation of exosomes from DCs culture medium. (**A**) The ultrastructure of exosome by transmission electron microscopy. Bar size, 100 nm. (**B**) The size distribution profile of immature and mature DC-exos by Nanosight, revealing a size peak of 113 nm in immature DC-exos and 109 nm in mature DC-exos. (**C**) The expression of exosomes negative marker, Calnexin and positive markers, Alix, CD63 and TSG101. And also, the expression of CCR7 was detected in both cell lysis and exosomes lysis. A total of 20ug protein from DCs lysis and 5 ug protein from exosomes lysis was loaded into each lane. Full-length blots can be found in Supplemental Figures 3 and 4.

We isolated exosomes from culture medium of immature and mature DCs and observed the ultrastructure of exosome using transmission electron microscopy. To adjust the concentration of exosomes, we used 500 ul PBS to suspend exosomes isolated from 10 ml DC culture medium (10^7^ cells). The protein concentration was approximately 0.2 ug/ul, indicating about 100ug exosomal protein being extracted from 10 ml culture medium. As we previously reported, the concentration of immature DC-exos and mature DC-exos had no difference^17^. Electron microscopic analysis revealed a typical size of 30 to 100 nm in diameter (Fig. 1A). To further investigate the size distribution profile of immature and mature DC-exos, we performed a size detection using the Nanosight, revealing a size peak of 113 nm in immature DC-exos and 109 nm in mature DC-exos (Fig. 1B), which is consistent with previous reports^6^. Then the expression of exosomes markers, Alix, CD63 and TSG101, were confirmed by immunoblotting (Fig. 1C). To test the purity of exosomes, we also determined the expression of Calnexin, which is a negative marker of exosomes. CCR7 was also detected and the results showed an increased expression in both mature DCs and mature DC-exos compared with immature DCs and immature DC-exos. These data indicate a successful isolation of exosomes from culture medium.

### DC-exos activate immature DCs and CD4+ T cells *in vitro*

**Figure 2:**
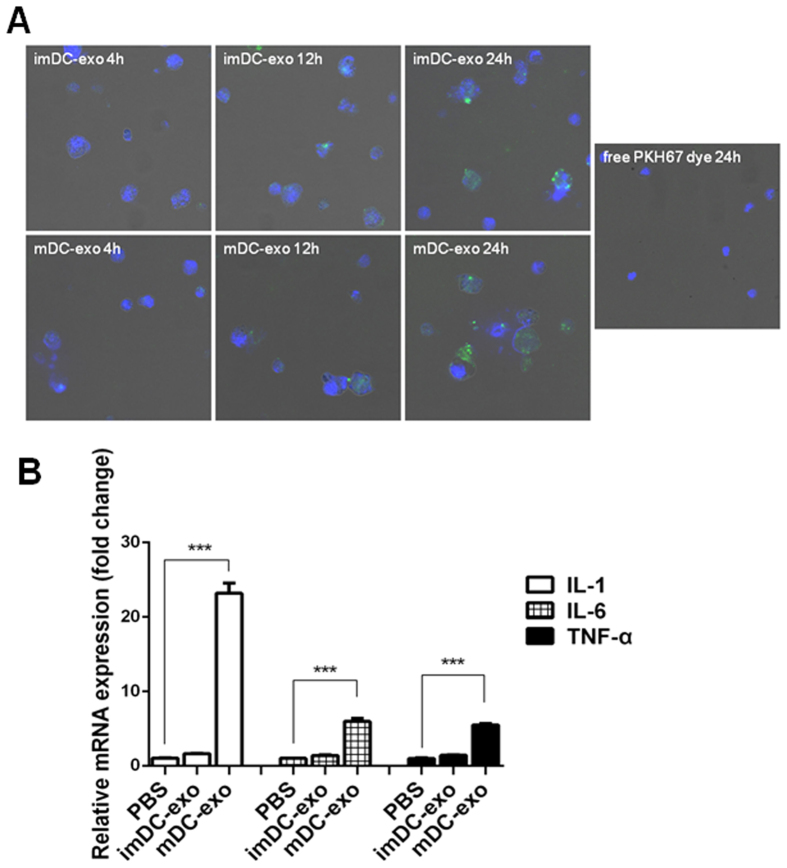
DC-exos uptake by DCs and inducement of inflammation *in vitro*. (**A**) The PKH67-labelled (green) immature DC-exos and mature DC-exos were co-cultured with immature DCs for indicated time. Fee PKH67 dye was also incubated with immature DCs as a positive control. Then the DCs were fixed and stained with DAPI (blue). The uptake of DC-exos by DCs was observed under a confocal microscope. (**B**) Immature and mature DC-exos were co-cultured with immature DCs for 24 hours. Then the DCs were harvested for the detection of IL-1, IL-6 and TNF-α by quantitive PCR (n = 3). ***p < 0.001.

The isolated immature and mature DC-exos were stained with PKH67 and added to immature DCs culture medium for indicated time. We observed that immature DCs could uptake both immature DC-exos and mature DC-exos in a time dependent manner (Fig. 2A). We then found that mature DC-exos significantly induced the expression of cytokines, IL-1, IL-6 and TNF-α, in immature DCs while immature DC-exos did not have this effect (Fig. 2B). These data indicate that mature DC-exos have the ability to activate standby immature DCs.

**Figure 3:**
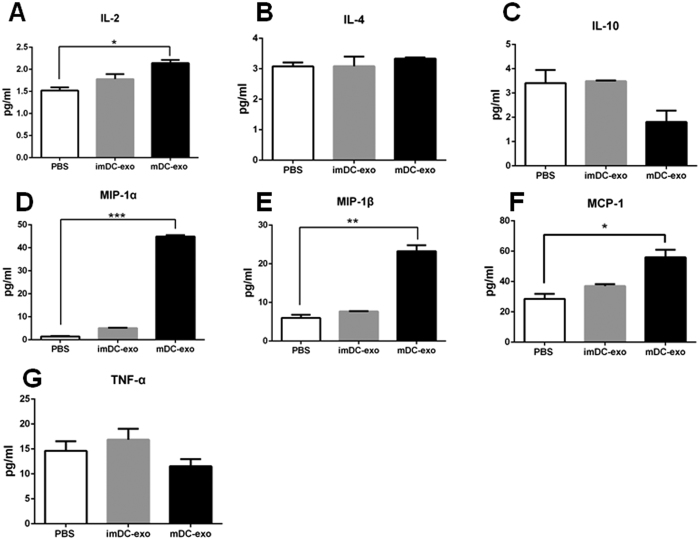
DC-exos activate CD4+ T cells *in vitro*. Immature and mature DC-exos were co-cultured with CD4+ T cells for 24 hours. Then the culture medium was collected for cytokines detection using a mouse inflammation kit (n = 4–5). *p < 0.05, **p < 0.01, ***p < 0.001.

As for CD4+ T cells, we previously demonstrated that the uptake of mature DC-exos was significantly higher than that of immature DC-exos^17^. Then we investigated the effects of both immature DC-exos and mature DC-exos on CD4+ T cells. We found that mature DC-exos increased the expression of IL-2, MIP-1α, MIP-1β and MCP-1 (Fig. 3A–G). However, we did not observe an elevation of TNF-α.

### Biodistribution of exosomes in mice

**Figure 4:**
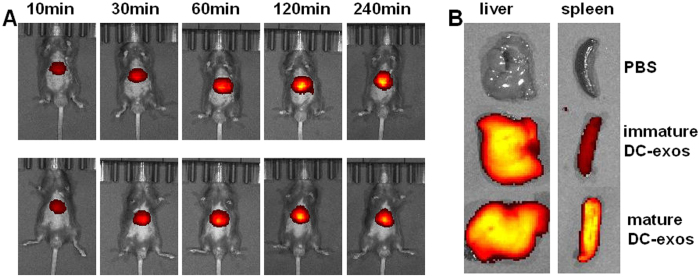
DC-exos accumulation in mice. (**A**) The DiR-labelled immature and mature DC-exos were intravenously injected into mice and then these mice were imaged by IVIS at indicated time. (**B**) Four hours after injection, the mice were sacrificed and livers and spleens were taken out for imaging. Representative images of 3 dependent studies.

To investigate the biodistribution of DC-exos, we stained exosomes with DiR and injected them into mice through tail vein. The mice were imaged using the IVIS at indicated time and we observed that both immature and mature DC-exos rapidly accumulated to upper abdomen within 4 hours (Fig. 4A). This result was consistent with a previous study^12^. Then the liver and spleen were taken out and observed. We found that mature DC-exo accumulated more in spleen compared with immature DC-exos (Fig. 4B). To investigate whether free DiR dye can accumulate in spleen, we injected both PBS and free DiR dye into mice and found that the free DiR dye mainly accumulated in liver (Supplemental Figure 1). Previous study has demonstrated that, no matter what cell types the exosomes derived, they mainly accumulated in liver^12^. We observed this phenomen too and further found that DiR could also accumulate in liver. It might be due to the metabolic function of liver. These data indicate the different ability of immature and mature DC-exos to accumulate in spleen.

### DC-exos migrate to white pulp and induce inflammation *in vivo*

**Figure 5:**
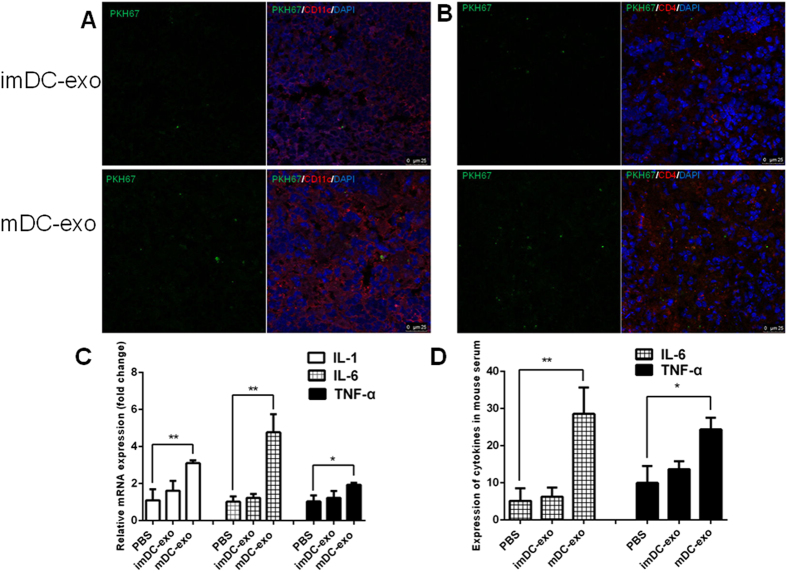
DC-exos uptake by splenetic DCs and T cells and inducement of inflammation *in vivo*. (**A**,**B**) The PKH67-labelled immature and mature DC-exos were intravenously injected into mice. After 24 hours, the mouse was sacrificed and the spleen was cut into 6um sections for staining CD11c (**A**) and CD4 (**B**) and DAPI. The uptake of DC-exos by splenetic DCs and T cells was observed under a confocal microscope. Representative images of 3 dependent studies. (**C**) Twenty-four hours after PBS or DC-exos injection, the spleen was harvested and the tissue total RNA was extracted for detection of IL-1, IL-6 and TNF-α by quantitive PCR (n = 4–5). (**D**) Twenty-four hours after PBS or DC-exos injection, the serum was collected for detection of IL-6 and TNF-α by Elisa (n = 5–6). *p < 0.05, **p < 0.01, ***p < 0.001.

Spleen is a secondary lymphoid organ and contains various immune cells. We investigated whether splenetic DCs and CD4 T cells could uptake the injected DC-exos. The results showed that both DCs and T cells in white pulp could indeed uptake exosomes (Fig. 5A,B). And the results also confirmed the increased accumulation of mature DC-exos than immature DC-exos. The accumulated exosomes in red pulp were much less than that in white pulp (Supplemental Figure 2). Since mature DC-exos could activate immature DCs and CD4+ T cells *in vitro* and, according to previous findings, activate T cells *in vivo*, we detected the expression of cytokines and found that mature DC-exos significantly induced inflammation in mice (Fig. 5C,D). These data indicate the ability of mature DC-exos to induce inflammation *in vivo*.

### CCR7 regulates DC-exos migration and inflammation in mice

**Figure 6:**
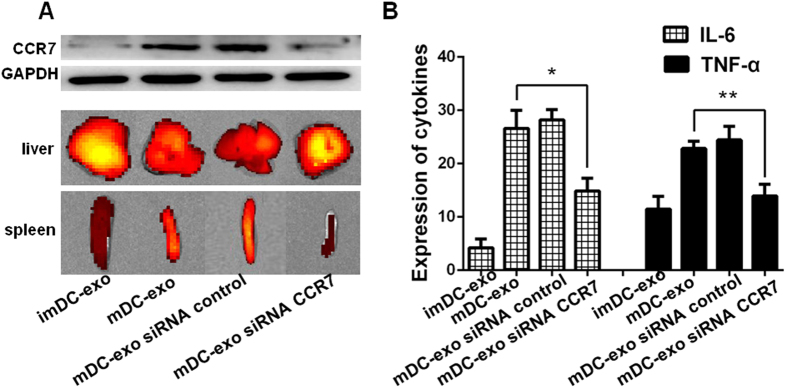
The role of CCR7 in mature DC-exos accumulation in spleen and inducement of inflammation *in vivo*. (**A** upper) The expression of CCR7 in immature DC-exos and mature DC-exos. When we used siRNA to down-regulate mature DCs’ expression of CCR7, its expression in mature DC-exos was also down-regulated. A total of 10ug exosomal protein was loaded into every lane. (**A** lower) The accumulation of immature DC-exos, mature DC-exos and mature DC-exos with down-regulated CCR7 in liver and spleen. Representative images of 3 dependent studies. (**B**) Immature DC-exos, mature DC-exos and mature DC-exos with down-regulated CCR7 were injected to mice. After 24 hours, the serum was collected for detection of IL-6 and TNF-α by Elisa (n = 5–6). *p < 0.05, **p < 0.01, ***p < 0.001.

The ability of mature DC-exos to accumulate in spleen is very similar to their parent DCs, which home to secondary lymphoid organs through CCR7. Since CCR7 was found increased in mature DC-exos, we postulated that the elevated accumulation of mature DC-exos in spleen might be due to CCR7. Indeed, when we down-regulated CCR7 in mature DC-exos (Fig. 6A upper), we found that their accumulation in spleen was also down-regulated (Fig. 6A lower). Finally, we showed that the expression of cytokines in mice serum dropped significantly when CCR7 in mature DC-exos was down-regulated (Fig. 6B).

## Discussion

Extracellular vesicles (EVs) have attracted a huge attention during the past decades. Exosomes belong to extracellular vesicles and they are formed within the endosomal network and released upon fusion of multi-vesicular bodies with the plasma membrane. Exosomes can be isolated from most cell types and biological fluids such as saliva, urine, nasal and bronchial lavage fluid, amniotic fluid, breast milk, plasma, serum and seminal fluid^5^. Upon release from parent cells, exosomes can stay among the microenvironment in organs or move to distant parts of the body, where they directly interact with target cells by membrane proteins or indirectly influence the function of cells by shuttling miRNAs to recipient cells. Thus researchers have spent lots of efforts to investigate the protein and miRNAs composition in exosomes.

As for DCs-derived exosomes, they carry surface MHC class I and class II molecules and, therefore, potentially can directly stimulate CD8^+^ and CD4^+^ T cells, respectively. Indeed, injection of DC-exos induced antigen-specific naïve CD4^+^ T cell activation *in vivo*. In the present study, although we found that T cells stimulated by mDC-exos expressed more IL-2, MIP and MCP, TNF-α remained unchanged. This finding was consistent with a previous study, which reported that, *in vitro*, DC-exos did not induce antigen-dependent T cell stimulation unless mature CD8α^−^ DCs were also present in the cultures^18^. This might be explained by that T cell stimulatory activity of exosomes was 10–20-fold less efficient than that of the parent cells^19^,^20^,^21^. Besides the interaction of DC-exos and T cells, DC-exos can also affect their parent DCs. Mature DC-exo could stimulate mature DCs and make the latter express a higher level of pMHC I, MHC II, and co-stimulatory CD40, CD54 and CD80^22^. Both immature and mature DC-exos could bind LPS and acquire the ability to strongly activate bystander immature DCs^23^. However, immature DC-exos alone could not activate immature DCs^23^. Here in the present study, we confirmed that immature DC-exos alone could not activate immature DCs. However, mature DC-exos alone could activate immature DCs. When intravenously injected to mice, DC-exos were observed to accumulate in liver and spleen and were up-taken by splenetic CD11c^+^ DCs and CD4^+^ T cells. The *in vivo* effect of DC-exos was demonstrated by elevated concentration of cytokines in serum. These results, combined with previous findings, are interesting because DC-exos could act just like DCs. They alert bystander DCs, activate T cells, migrate to lymphoid organs and induce inflammation.

Previous studies indicated that the half-life of purified exogenous EVs, artificially introduced into circulation, is very short, ranging from about 10 minutes to 5 hours^24^,^25^,^26^,^27^,^28^. It is believed that the clearance of EVs from circulation is most likely due to uptake in target organs and the distribution most probably depends on the parent cell source, as well as the availability of different target cell types to internalize the circulating EVs. Indeed, the majority of EVs derived from red blood cell are taken up by liver and bone^28^ and exosomes derived from melanoma are mainly taken up by lungs and spleen^25^. Most recently, an *in vivo* study investigated the biodistribution of exosomes derived from DCs^12^. The researchers founded that, compared with exosomes derived from muscle cells and melanoma cells, DC-exos showed increased accumulation in spleen. Lyden and his colleagues have made a milestone on exploring why and how exosomes migrate to different organs after injection to mice^29^. They found that exosomes from MDA-MB-231 cell line, which metastasize primarily to the lung, accumulated more in lung and exosomes from BxPC-3 and HPAF-II cell lines, which metastasize primarily to the liver, accumulated more in liver. The authors then demonstrated that different exosomes express different integrins, which orchestrate the accumulation of exosomes in specific organs. These results showed a potential relationship between parent cells and their exosomes in regarding to the exosomes migration *in vivo*. Here in the present study, we observed an increased accumulation of mature DC-exos in spleen than immature DC-exos.

CCR7 has been demonstrated to be involved in DCs homing and, along with other co-stimulatory molecules, it is highly expressed when immature DCs go mature. CCL19 and CCL21 are the sole ligands for the CCR7. CCL21a is mainly expressed in secondary lymphoid organs and CCL21b is expressed in peripheral tissues. In contrast, CCL19 is restricted to the thymus and secondary lymphoid organs. CCR7 and its ligands are essentially involved in homing of T cells and DCs to the lymph organs^2^.

An early study found an expression of CCR7 in mature DC-exos, although the expression is relatively lower than that in mature DCs^22^. That study did not investigate the differential expression of CCR7 in immature DC-exos and mature DC-exos. In this present study, we confirmed the expression of CCR7 in DC-exos. And we also showed that its expression in mature DC-exos was higher than immature DC-exos. This finding can explain the different accumulation of immature DC-exos and mature DC-exos in spleen. Indeed, the accumulation of mature DC-exos in spleen was significantly less when we down-regulated CCR7. And as a result, the concentration of cytokines in serum synchronously declined.

There were several limitations in this study. To obtain exosomes, we used the ExoQuick-TC, which achieves high yield but a low specificity of exosomes. Our electron microscopic images showed blurred background and they were not as perfect as that published in previous study. We recommend that a better way to isolate exosomes should be used in future studies.

In conclusion, our study suggests that exosomes derived from mature DCs can activate immature DC and T cells *in vitro*, accumulate in spleen and induce inflammation *in vivo*, which is regulated by CCR7. This finding provides us a new perspective to comprehensively understand exosomes, which might inherit some special functions from their parent cells and exert these functions *in vivo*.

## Additional Information

**How to cite this article:** Wei, G. *et al*. Dendritic cells derived exosomes migration to spleen and induction of inflammation are regulated by CCR7. *Sci. Rep.* **7**, 42996; doi: 10.1038/srep42996 (2017).

**Publisher's note:** Springer Nature remains neutral with regard to jurisdictional claims in published maps and institutional affiliations.

## Supplementary Material

Supplementary Data
