# Supplementary material for: Dendritic cells derived exosomes migration to spleen and induction of inflammation are regulated by CCR7
Source: Sci Rep. 2017 Feb 22;7:42996. doi: 10.1038/srep42996 (PMC5320445; doi:10.1038/srep42996)
Supplement: Supplementary Data [file srep42996-s1.doc]

**Dendritic cells derived exosomes migration to spleen and induction of inflammation are regulated by CCR7**

Gao Wei, Yuan Jie, Liu Haibo, Wu Chaoneng, Huang Dong, Zhu Jianbing, Guo Junjie, Ma Leilei, Shi Hongtao, Zou Yunzeng, Ge Junbo

Supplemental Figure 1


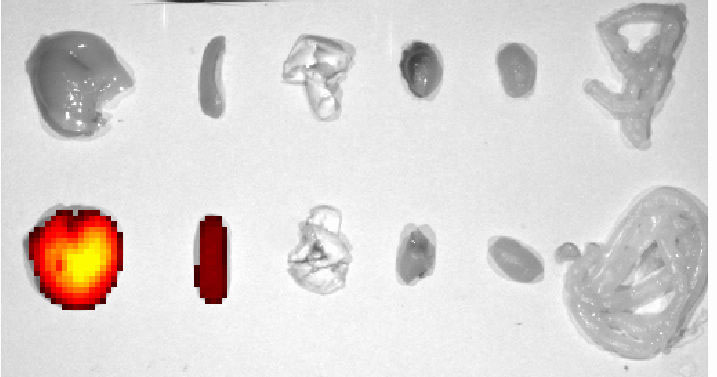


A total of 100ul PBS with or without 5ul DiR was injected into mice and organs were taken out for imaging after 4 hours. Upper, 100ul PBS. Lower, 100ul PBS with 5ul DiR. From left to right, the organs are liver, spleen, lung, heart, kidney and small intestine, respectively.


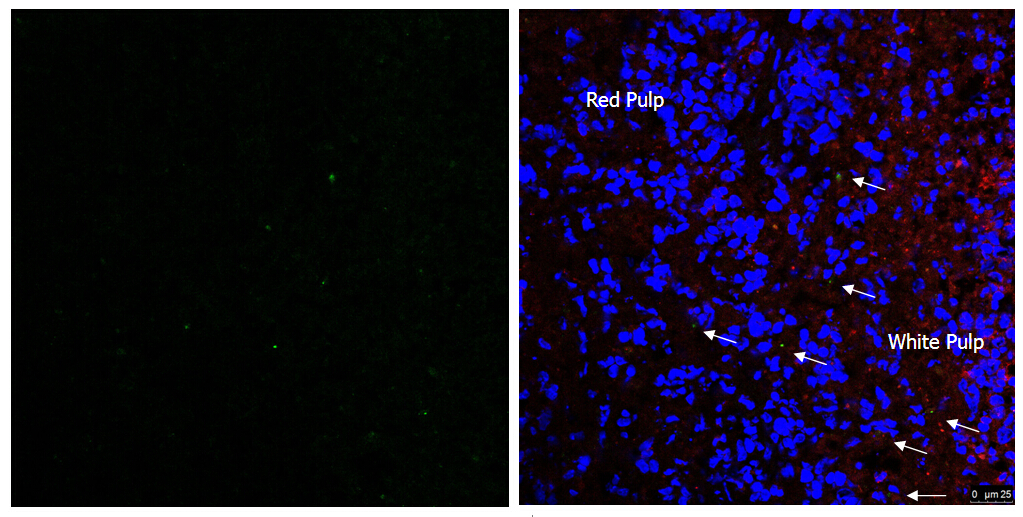
Supplemental Figure 2

**A**


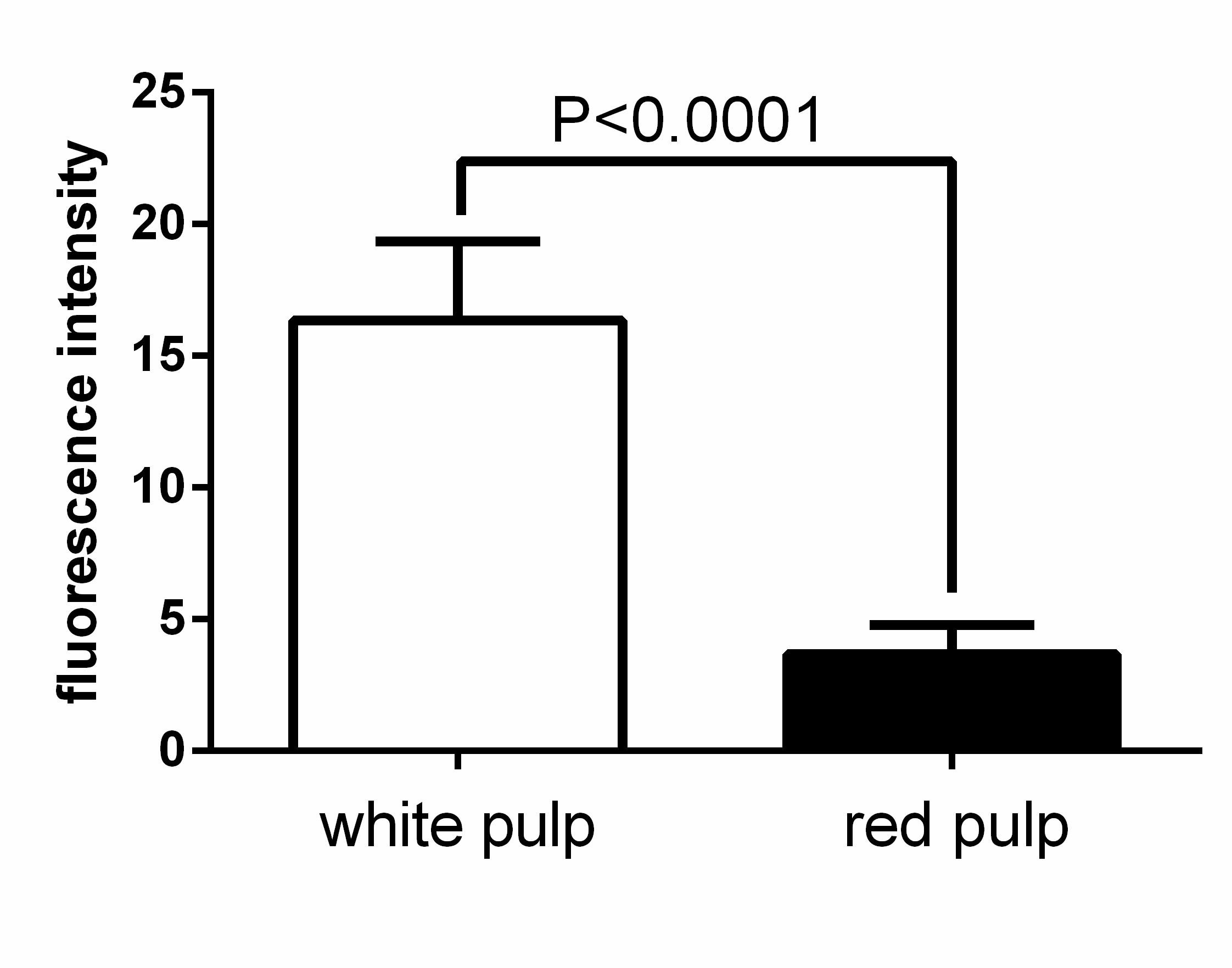
**B**

(A) Left, PKH67 layer showing the accumulation of exosomes (green) in spleen. Right, composite layer showing CD4 (red), nuclei (blue) and exosomes (green). (B) Fluorescence intensity of PKH67 labeled exosomes in white and red pulp. Data represent the mean ± SD from 9 images of three mice.

Supplemental Figure 3


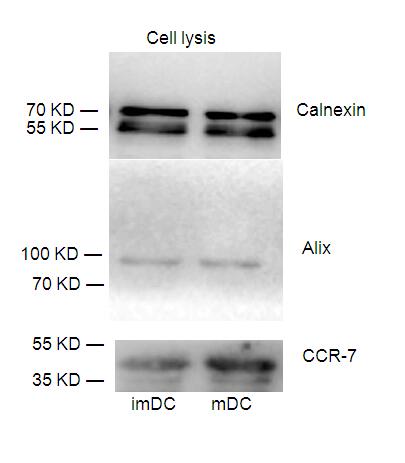


Full-length blots of Calnexin, Alix and CCR-7 in cell lysis were shown. For Calnexin, we can see a non-specific banding around 55 KD. We have repeated 3 times and this banding can be persistently detected.

Supplemental Figure 4


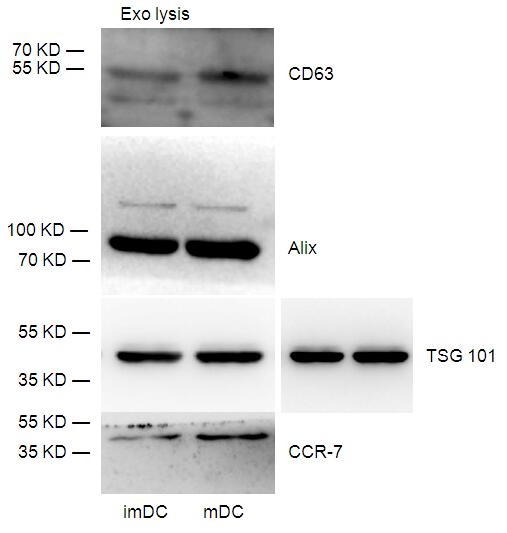


Full-length blots of CD63, Alix, TSG 101 and CCR-7 in exosomal lysis were shown. TSG 101 was of overexposure and we demonstrated two images with different sensitometry to display more details.
